# Social service offices as a point of entry into substance abuse treatment for poor South Africans

**DOI:** 10.1186/1747-597X-7-22

**Published:** 2012-05-29

**Authors:** Nadine Harker Burnhams, Siphokazi Dada, Bronwyn Myers

**Affiliations:** 1 Alcohol and Drug Abuse Research Unit, South African Medical Research Council, P.O. Box 19070, Tygerberg 7505, South Africa; 2 Department of Psychiatry and Mental Health, University of Cape Town, Tygerberg South Africa

**Keywords:** Substance abuse, Epidemiology, South Africa, Social services

## Abstract

**Background:**

In South Africa, district social service offices are often the first point of entry into the substance abuse treatment system. Despite this, little is known about the profile of people presenting with substance-related problems at these service points. This has a negative impact on treatment service planning. This paper begins to redress this gap through describing patterns of substance use and service needs among people using general social services in the Western Cape and comparing findings against the profile of persons attending specialist substance abuse treatment facilities in the region.

**Methods:**

As part of a standard client information system, an electronic questionnaire was completed for each person seeking social assistance. Data on socio-demographic characteristics, the range of presenting problems, patterns of substance use, perceived consequences of substance use, as well as types of services provided were analysed for the 691 social welfare clients who reported substance use between 2007 and 2009. These data were compared against clients attending substance abuse treatment centres during the same time period.

**Results:**

Findings indicate that social services offices are used as a way of accessing specialist services but are also used as a service point, especially by groups under-represented in the specialist treatment sector. Women, people from rural communities and people with alcohol-related problems are more likely to seek assistance at social service offices providing low threshold intervention services than from the specialist treatment sector.

**Conclusions:**

The study provides evidence that social services are a point of entry and intervention for people from underserved communities in the Western Cape. If these low-threshold services can be supported to provide good quality services, they may be an effective and efficient way of improving access to treatment in a context of limited service availability.

## Background

The provision of substance abuse services has become critical in South Africa given high lifetime population prevalence estimates of 13.3% for substance use disorders [1]. Although studies document high prevalence rates for South Africa as a whole, the Western Cape Province is particularly afflicted by substance abuse problems. For example, the first South African Stress and Health Study, a nationally representative study, found that the Western Cape had a significantly higher lifetime prevalence rate for substance abuse and dependence (18.5%) than the national average (13.3%) [2].

The burden that these disorders place on the health and welfare system of South Africa is compounded by high levels of unmet substance abuse treatment needs, particularly within poor South African communities [3]. To a large extent, the roots of these unmet treatment needs are located in the inequitable spread and limited availability of substance abuse treatment services across South African communities. This is mainly because race was a major determinant of access to health and social resources (including substance abuse treatment) in apartheid South Africa [4,5], with White South Africans having better access to public services than Coloured (people of mixed race ancestry) or Black African South Africans. During this era, public services were concentrated in urban areas reserved for the use of White South Africans [6]; with the racial segregation of public services limiting the use of available services by Black African and Coloured South Africans [4,5]. Geographical apartheid contributed to poor access to services and poverty, with Black African and Coloured South Africans forced to live in peri-urban township areas (with few economic opportunities) located considerable distances from White, economically advantaged, urban areas [4,5,7].

After 18 years of democracy, South Africa is still grappling with the legacy of apartheid (including less access to economic opportunities and geographic apartheid for historically disadvantaged groups), and the challenges of transforming and promoting equitable access to public services [7], including substance abuse treatment. Despite the availability of a diverse network of specialist substance abuse treatment facilities in the Western Cape (that includes stand-alone inpatient (residential) as well as outpatient services) dedicated to the treatment of substance use disorders [8], these services remain difficult to access for poor Black African and Coloured South Africans who, for the most part, continue to reside in townships that have few services, poor infrastructure and limited economic opportunities. These residual spatial inequalities [9] contribute to poor South Africans having limited access to specialised substance abuse services, with the geographical location of existing services, the costs associated with travelling to distant services, and the costs associated with paying for treatment in a system where there are few free services available being major barriers to treatment entry [10].

However specialist substance abuse treatment facilities are not the only providers of substance abuse intervention services in the country. In an effort to improve the availability of drug treatment services in the Western Cape, the state has increased resources for the provision of substance abuse early intervention, referral, and aftercare services at district social service offices [6]. These general social service offices provide a broad range of social welfare services that include counselling and assistance for family problems; with social grants (poverty relief), child welfare and custody issues; and assessment, early interventions for substance-related problems, and referral where needed to more specialist substance abuse treatment services [6]. It should be noted that the substance abuse services provided by these offices differ from those offered by specialist substance abuse treatment facilities in that services provided are typically of a shorter duration, are provided by generalist social workers without a specialist interest in substance use disorders, and do not involve the provision of psychosocial and behavioural treatment for substance dependence [8]. Despite these differences, for disadvantaged communities in the Western Cape, state social work offices that provide social welfare services are often the first port of call when seeking assistance for substance-related problems [11]. This is partly because services are free and also because these services are located within every district of the province and are within easy reach of poor communities.

Despite this, little is known about the profile of persons presenting with substance-related problems at these service points. This is worrisome as these data are an important additional source of information on patterns of substance use and associated treatment needs in the province. At present, the South African Community Epidemiology Network on Drug Use (SACENDU) project [12] is the only routine source of data used to assess treatment demand. SACENDU is a network of researchers, practitioners and policy makers from five sentinel areas in South Africa. Since 1996, this project has collected, on a six-monthly basis, descriptive information on the socio-demographic and substance use profile of all clients served at inpatient and outpatient substance abuse treatment centres in South Africa [12]. While the SACENDU project provides essential information that should be collected as part of any substance abuse surveillance system, this data source has some inherent disadvantages including only reflecting service needs among those people who are able to access specialist treatment services [11]. This paper hopes to redress this gap through describing key findings from an additional substance abuse surveillance system located within the social welfare service system. This data system is potentially useful as it facilitates the triangulation of data from multiple sources, thereby potentially strengthening our understanding of treatment needs in the province. This paper aims to support the triangulation of data on patterns of substance use in the Western Cape through describing the demographic and substance use profiles of persons seeking help for substance-related problems at social welfare offices in the Western Cape between November 2007 and December 2009 and comparing these findings against those from the SACENDU project.

## Method

In 2007, an electronic substance abuse surveillance system (SASS) for district social welfare offices in the Western Cape was implemented. SASS was developed to complement and strengthen the existing SACENDU system (that only collects substance abuse information from specialist substance abuse treatment facilities [12]) through describing the nature and extent of substance abuse and (unmet) service needs among people seeking social welfare services in the Western Cape Province [11]. The design and implementation of this system has been described at length elsewhere [11].

Social workers responsible for intake assessments at each of the 16 social welfare districts in the Western Cape use the SASS to routinely collect data on substance use, problems associated with substance use, and service needs from all persons seeking social welfare assistance; regardless of their substance use status. For the purpose of this paper, we will present SASS data only for the 691 people who accessed the social welfare system between November 2007 and December 2009 and reported using alcohol or other drugs. These individuals represent 38% of the total proportion of clients seeking social welfare services for which data collection forms were completed. Data were collected from 16 welfare districts in the Western Cape (Table 1).

**Table 1:** Proportion of substance use cases reported at participating district social service offices (November 2007-December 2009)

**District**	**Rural/Urban district**	**N**	**%**
Athlone District Office	Urban	59	8.5
Beaufort West District Office	Rural	154	22.3
Bellville District Office	Urban	32	4.6
Caledon District Office	Rural	22	3.2
Cape Town District Office	Urban	1	<1
Eerste river District Office	Urban	65	9.4
George District Office	Rural	40	5.8
Gugulethu District Office	Urban	6	1
Khayelitsha District Office	Urban	-	-
Mitchell’s Plain District Office	Urban	62	9
Oudtshoorn District Office	Rural	62	9
Paarl District Office	Urban	3	<1
Vredenburg District office	Rural	32	4.6
Vredendal District Office	Rural	65	9.4
Worcester District Office	Rural	73	10.6
Wynberg District Office	Urban	15	2.2

In contrast, the SACENDU project collects data on all patients admitted to specialist substance abuse treatment services across five provincial sites on a six monthly basis [12]. For this system, treatment providers at participating facilities complete a one page form detailing the demographic characteristics of the patient, patterns of substance use and treatment history. In this paper, we report on data for the 17,631 people who were admitted to specialist treatment centres in the Western Cape between November 2007 and December 2009 in the Western Cape. Both the SACENDU and SASS system, ethical approval was granted by the University of Stellenbosch’s Health Research Ethics Committee.

### Measures

For the SASS system, data are collected using an electronic questionnaire that is completed for each person seeking social welfare assistance. The form consists of 27 forced-choice questions and takes approximately 5–10 minutes to complete. This questionnaire elicits responses about socio-demographic characteristics, the range of presenting social welfare problems, patterns of substance use, perceived (subjective) consequences of substance use, as well as types of services provided by the social worker to each client. The items contained in the form were taken from the SACENDU data collection tool [12] as well as the Treatment Services Audit questionnaire, used to audit substance abuse treatment services in South Africa [13]. More specifically, the SASS data collection form contains the following variables:

#### Socio-demographic characteristics of the client

These items were taken from the SACENDU data collection form and included forced-choice items pertaining to the age, gender, race/ethnicity (Black African, Coloured, Asian/Indian or White), and suburb of residence of the client as well as the source of referral to social welfare services (self, family/friends, employer, NGO, health professional, religious group, school, courts/correctional services). Other items examined the highest level of education completed, the employment status of the client, and the current marital status of the client.

#### Range of presenting social welfare problems

This item asked about the reason for seeking social welfare services. Possible reasons for seeking social services included: family problems, work problems, financial problems, social security needs, child abuse/neglect issues, problems related to the use of alcohol or drugs, domestic violence problems, need for statutory services (such as probation, foster care needs), child custody issues, housing needs (including homelessness), and family members with substance abuse problems. This item was not included in the SACENDU data collection form.

#### Patterns of substance use

Several items examined the client’s use of alcohol and other drugs. These items were taken directly from the SACENDU data collection form. The first item was a filter question which asked about the current (past month) use of alcohol or drugs. If the person reported the absence of current substance use, no further questions were asked. Those clients who reported the current use of substances were asked about the types of substances used. For each reported substance, clients were also asked about the frequency with which they used this substance (daily, 2–6 times per week, once per week or less often, or not used in the past month), and the age at which they first started using this substance. Clients who reported the use of heroin were also asked whether they had injected this drug in the six months prior to the interview.

#### Perceived problems associated with substance use

One item examined problems associated with the client’s use of alcohol and other drugs. Response options included: family problems, scholastic problems, expulsion or suspension from school, suspension from work, health problems, financial problems, trouble with the law, marital or relationship problems, domestic violence, and other problems. For the “other problems” category, participants were asked to specify these problems. This item was not collected by the SACENDU data collection form.

#### Previous treatment history

The SASS data collection form included items that referred to previous substance abuse treatment history. Similar to SACENDU, clients were asked about whether they had ever received treatment for substance abuse problems (yes/no). The SASS data collection sheet also collected additional information on treatment history that was extracted from the Treatment Audit questionnaire [13]. First, clients were asked to list the type of treatment they received in their last treatment episode. Options for this item included inpatient treatment, outpatient treatment, detoxification only, involuntary committal, general counselling services, or other type of treatment. Finally, clients were asked whether they had completed their previous treatment episode (yes/no).

#### Type of services provided by social workers

Two items examined the types of services provided to clients. The first item asked about the type/s of services provided during the intake process. Possible services that clients received at intake included: crisis intervention, counselling, social grant, assessment, housing placement, custody-related services or referral to additional services. The second item examined the types of additional services to which clients were referred. Here response options included referrals to inpatient substance abuse treatment, outpatient substance abuse treatment, substance abuse support groups (AA), involuntary committal to treatment, other welfare services, nongovernment organizations, psychiatric assessments, detoxification, crisis intervention services or the case was closed.

### Data analysis

Statistics for this study were computed using the Statistical Package for the Social Sciences (version 18). First we combined the SASS and SACENDU data sets for the periods 2007–2009 into a single data set. Descriptive statistics were calculated for all variables in this combined data set. In addition, we performed Chi square tests of association for categorical variables and paired sample t-tests for continuous variables to determine whether there were significant differences in the socio-demographic profile and pattern of substance use between persons presenting for services by data source (SASS versus SACENDU).

## Results

### Demographic profile of substance-using and non-using persons seeking social welfare services

For the SASS database, two-thirds of social welfare clients who reported substance use were male. These persons were mainly Coloured (85.9%) with Black African persons comprising 11.4% of the sample (Table 2). Social welfare clients reporting substance use were relatively young, with the mean age of the sample being 25.2 years (SD = 11.98) and 42.4% of the sample being between 15 and 19 years of age. Just over half of the participants were unemployed and only 23.6% were in any form of employment (Table 2). Compared to people who reported substance use, a higher proportion of clients who reported not using substances were female (61.8% vs 33.2%), Black African (29.0% vs 11.4%) and older; with the average age of non-substance using clients being 33.7 years of age (SD = 16.8; Table 2).

**Table 2:** Population profile of substance-using and non-substance using persons within the SASS data set compared with the SACENDU data collected between 2007–2009)

	**SASS data (1817 cases)**				**SACENDU data**		**Comparison between substance users in SASS and SACENDU**	
	**Substance users (691)**		**Non-substance users (1126)**		**Substance users only (17631)**		**Test statistic (df)**	**P value**
	**N**	**%**	**N**	**%**	**N**	**%**		
**Gender**								
Male	459	66.8	430	38.2	13197	74.9	21.92(1)	<0.001
Female	228	33.2	696	61.8	4434	25.1	21.93 (1)	<0.001
**Race**								
Black African	78	11.4	326	29.0	1529	8.7	5.12 (1)	0.02
Asian/Indian	2	<1	26	2.3	132	<1	1.65 (1)	0.19
Coloured	590	85.6	753	67.0	12279	69.9	68.45 (1)	<0.001
White	17	2.5	19	1.7	3620	20.6	120.69(1)	<0.001
**Mean age (years, SD)**	25.21	11.98	33.7	16.8	28.11	11.56	6.44 (16773)	<0.001
**Employment**								
Working	163	23.6	290	25.8	4973	31.0	16.75 (1)	<0.001
Unemployed	365	52.8	468	41.7	8272	51.5	.520 (1)	0.47
Student/learner	138	20.0	181	16.1	2177	13.6	23.09 (1)	<0.001
Housewife	7	1.0	54	4.8	108	0.7	1.14 (1)	0.29
**Marital status**								
Married, living with spouse	78	11.3	313	29.7	3139	19.5	28.81 (1)	<0.001
Living together	32	4.6	78	7.0	950	5.9	1.97 (1)	0.34
Divorced	67	9.7	72	6.4	976	6.1	15.03 (1)	<0.001
Single	493	71.4	532	47.4	10763	66.9	6.11 (1)	0.01
**Education**								
Primary (Grade 1–7)	279	41.1	197	17.9	2076	13.2	414.20 (1)	<0.001
Secondary (Grade 8–12)	379	55.8	604	53.6	12094	76.7	153.99 (1)	<0.001
Tertiary	0	0.0	300	26.6	1538	9.7	73.01(1)	<0.001
**Primary substance of abuse**								
Alcohol	322	46.6	−	−	5075	31.5	69.54 (1)	<0.001
Cannabis	190	27.5	−	−	2309	14.3	90.92 (1)	<0.001
Methamphetamine	132	19.1	−	−	6623	41.1	133.10 (1)	<0.001
**Presenting problems**								
Family problems	274	39.7	336	29.8	−	−		
Work	74	10.7	21	1.9	−	−		
Financial	181	26.2	194	17.2	−	−		
Social security	27	3.9	70	6.2	−	−		
Child neglect/abuse	69	10.0	123	10.9	−	−		
Substance abuse problems	410	59.3	68	6.0	−	−		
Domestic violence	118	17.1	63	5.6	−	−		
Statutory intervention	113	16.4	310	27.5	−	−		
Custodial issues	20	2.9	89	7.9	−	−		
Homeless/Destitute	19	2.7	34	3.0	−	−		
Substance abuse in the family	115	16.6	94	8.3	−	−		

### Comparison of the demographic profile of substance-using persons seeking social welfare services and specialist substance abuse treatment services

When we compared the profile of substance-using clients with the profile of clients seeking specialist substance abuse treatment (in the SACENDU data set), several differences were found (Table 2). The SASS data set had a significantly higher proportion of women compared to the SACENDU system (Chi-square = 21.92 (df = 1), *p* < 0.001). In terms of race, significantly more Black African (Chi-square = 5.12 (df = 1), *p* < 0.02) and Coloured clients (Chi-square = 68.45 (df = 1), *p* < 0.001) sought services at social welfare offices compared to specialist treatment facilities. In addition, the substance-using clients seeking social welfare services were significantly younger than those attending specialist drug treatment facilities (*t* = 6.44 (df = 16773), *p* < 0.001). A significantly smaller proportion of substance-using clients attending social welfare services were employed compared to those attending specialist treatment facilities (Chi-square = 16.75 (df = 1), *p* < 0.001). In addition, a significantly smaller proportion of clients attending social welfare services were married (Chi-square = 28.81 (df = 1), *p* < 0.001) compared to clients from the SACENDU dataset (Table 2). Finally clients from the SASS system were significantly less educated than clients from the SACENDU system; with a significantly greater proportion of clients from the SASS system not completing high school compared to clients from the SACENDU database (Chi-square = 414.20 (df = 1), *p* < 0.001).

### Presenting problems among substance-using and non-using persons seeking social welfare services

Among clients who reported substance use, the most frequently reported reason for using social services was “substance-related problems”, for which 59.3% requested assistance (Table 2). Following this, the second and third most frequently reported reason for seeking services was family and financial problems, with 39.7% and 26.2% of the clients who used substances citing these problems respectively. A further 17.1% and 16.6% presented with problems related to domestic violence and difficulties associated with family members who had substance abuse problems respectively (Table 2). In contrast, the three most frequently reported reasons for seeking social services among clients who reported not using substances were family problems, statutory interventions, and financial problems; with 29.8%, 27.5% and 17.2% of the non-substance using clients reporting these problems respectively.

### Comparison of substance use patterns among people seeking social welfare and specialist substance abuse treatment services

Among the 691 social welfare clients who reported substance use, alcohol was the most frequently reported primary substance of abuse. While close to 50% of these clients reported the use of alcohol (Table 2), most drank alcohol once a week or less often, 32.7% of drinkers drank on two to six days of the week, and only 13.1% drank every day. Cannabis was the second most frequently reported substance of use (with 27.5% of the 691 clients reporting the use of this drug), followed by methamphetamine which was used by 19.1% of the sample (Table 2). Among people who smoked cannabis, the majority (49.5%) reported smoking it daily and 29.3% reported smoking it two to six times per week. For people who reported the use of methamphetamine, most (43.9%) reported using this drug two to six times per week, while 41.7% reported using it on a daily basis. Only 12.9% (n = 89) of clients had previously received treatment for a substance use disorder. Of these 89 people, only 50.6% (n = 45) had completed their last treatment episode.

In contrast, among people accessing specialist treatment centres, methamphetamine was the most frequently reported substance of abuse with 41.1% reporting this as their primary drug of abuse. A significantly greater proportion of clients from specialist substance abuse treatment facilities reported the use of methamphetamine than those from social welfare services (Chi-square = 133.10 (df = 1), *p* < 0.001). More than half (53%) of these clients reported the daily use of methamphetamine. Alcohol was the primary substance of abuse for 31.5% of clients attending specialist treatment facilities, with the majority (61.0%) of these clients reporting daily alcohol use. However, a significantly smaller proportion of clients from specialist substance abuse treatment facilities reported alcohol as their primary substance of abuse compared with substance-using clients attending social welfare services (Chi-square = 69.54 (df = 1), *p* < 0.001). Cannabis was reported as a primary drug of abuse by 14.3% of clients admitted to specialist treatment facilities. A significantly smaller proportion of clients from specialist substance abuse treatment facilities reported cannabis as their primary substance of abuse compared with substance-using clients attending social welfare services (Chi-square = 90.92 (df = 1), *p* < 0.001).

### Rural–urban comparisons for primary substance of abuse among people seeking social welfare services

Close to two-thirds of substance-using persons seeking social welfare services were from rural districts (Table 1). More than half of the substance-using persons from rural districts reported the use of alcohol (57.0%), with 21.5% reporting the use of cannabis and 16.2% reporting methamphetamine as their primary drug of choice. In contrast, only 27.2% of substance-using persons from urban districts reported alcohol as their substance of choice. In the urban districts, 38.5% of substance using clients reported cannabis and 24.7% reported methamphetamine as their primary drug of choice respectively.

### Problems associated with substance use for social welfare clients

**Figure 1:**
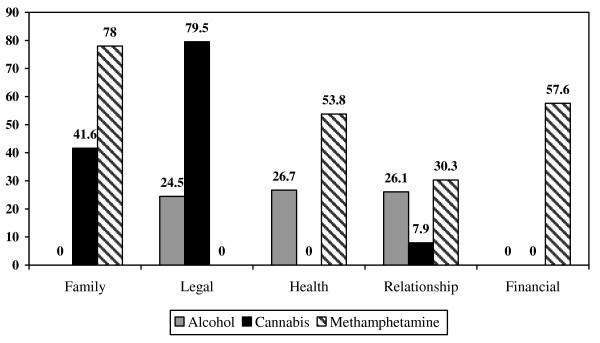
Proportion (%) of substance-using clients at district social service offices reporting various problems associated with substance use.

Family, legal, financial, health and domestic violence problems were the five most frequently reported problems associated with substance use, with 57.2%, 45.4%, 35.9%, 33.6% and 29.7% of the sample reporting these associated problems, respectively. A larger proportion of participants who used methamphetamine reported family, health, marital and relationship, and financial problems associated with their substance use compared to people who used cannabis or alcohol (Figure 1).

Specifically, clients who used methamphetamine were about three times more likely to report family (Chi-square = 29.02, df = 1, *p* < 0.001; OR 3.25, 95% CI = 2.08–5.06), financial (Chi-square = 33.35, df = 1, *p* < 0.001; OR 3.05, 95% CI 2.06–4.50), and health problems (Chi-square = 29.89, df = 1, *p* < 0.001; OR 2.87, 95% CI 1.95–4.24) and almost two times more likely to report marital/relationship problems (Chi-square = 7.94, df = 1, *p* < 0.001; OR 1.83, 95% CI 1.19–2.81) associated with their drug use than clients who did not use methamphetamine.

### Services provided to social welfare clients who reported substance use

The most common type of intervention received by clients during their first contact with social services was referral to specialist substance abuse treatment services for more intensive treatment. More than a third (35.9%) of substance-using clients received this service. The second and third most common types of interventions were once-off counselling services and brief interventions for substance use disorders; which 28.9% and 28.5% of the sample obtained, respectively. A further 21.7% of clients were referred for further assessment prior to making a determination about the types of intervention services required. Additional crisis intervention, placement and custody counselling services were provided to 10.4%, 2.3%, and 1.7% of social service clients, respectively.

## Discussion

Our findings suggest that general social services are an important point of entry into the substance abuse treatment system for people from socio-economically disadvantaged backgrounds. Within our sample, unmet service needs were high and only 13% of substance-using clients receiving social welfare services had received prior treatment for a substance use disorder. This is not surprising given the limited availability of substance abuse treatment in South Africa and the documented difficulties that people from disadvantaged communities have in accessing substance abuse treatment [3,6]. For treatment naïve individuals from disadvantaged communities, district social services appear to act as a bridge into substance abuse treatment, with more than a third of the substance-using clients in the SASS data system being referred to specialist substance abuse treatment services.

Apart from being an important entry point into substance abuse services for people from disadvantaged communities, our findings suggest that district social service offices are an important point of intervention for people with substance use disorders; with more than two-thirds of substance-using clients receiving some form of intervention for their substance-related problem. While the types of substance abuse intervention services provided by district social service offices are often low-threshold (comprising mainly of brief interventions or once-off counseling sessions), these services are helpful as they expand the range of substance-related services available in the province. Historically, the main type of intervention provided to people with substance use disorders has been inpatient (residential) or intensive outpatient treatment services provided by specialist substance abuse treatment agencies. These high-threshold services are costly to provide, time-intensive and difficult to access because of the limited availability of treatment slots [3,6]. Our finding that social workers within district social service offices not only refer clients to these high-threshold services but also provide lower threshold interventions is a promising development. If the policy environment continues to support investment in the provision of low threshold services, this may strengthen the existing substance abuse treatment system through reducing waiting lists for high threshold services and increasing the availability of substance abuse interventions in the province.

In addition, our findings hold value for service planning as they provide further insight into the typical profile of persons using alcohol and drugs in the Western Cape. Despite some similarities in the typical profile of person seeking substance abuse services (being male, Coloured, single and unemployed), several significant differences were found between clients within the SACENDU and SASS data systems. First, while men form the bulk of substance-using clients in both specialist substance abuse treatment and social welfare services, women were significantly more likely to seek substance abuse services from social welfare offices than specialist substance abuse treatment facilities. Specifically, women comprised more than a third of all substance-using clients at social service offices whereas women comprised only 20%–25% of admissions at specialist substance abuse treatment facilities [14]. One explanation for this difference may lie in findings from previous research which noted that women experience greater difficulties and more barriers in accessing specialist substance abuse treatment services compared to men [15]. It is quite possible that women seek assistance from district social service offices for their substance use as there are fewer barriers to accessing these services compared to specialist treatment facilities.

Another partial explanation for the large proportion of women in our study may lie in the fact that this sample was selected by screening all prospective users of general social services who did not necessarily report substance abuse as their presenting problem. For example, a third of the sample presented for help with a family problem and more than a quarter cited financial problems as their reason for seeking social welfare assistance. For many South African women, substance use remains hidden and they may be reluctant to seek substance abuse-related services due to the stigma associated with women who use substances [16]. This stigma partially arises from the perceived inability of substance-using women to fulfill traditional gender roles, such as taking care of dependent children [17]. As people from poor communities seek social welfare assistance for all kinds of reasons, there is probably little stigma associated with using general social services. As a result, women who are concerned about being stigmatised may find general social services more appealing than specialist substance abuse services. This claim is supported by evidence which shows that women are more likely to seek care for mental health or physical health problems (often related to their substance use) and to avoid seeking help for substance abuse [18]. These findings suggest that the routine screening of users of general social services for substance use provides social workers with an opportunity to intervene with individuals who may otherwise not have sought care for their substance use. This may allow for the early identification of people with substance-related problems and for interventions to occur at an earlier stage of problem severity before problems become entrenched and require intensive intervention services.

A further difference between the profile of substance-using clients attending general social welfare services and those attending specialist substance abuse treatment services is that clients from social welfare services appear to be more vulnerable. Specifically, general social welfare services served a significantly greater proportion of Black African and Coloured persons, younger, and poorly educated persons who were less likely to be employed than specialist substance abuse treatment facilities in the province. As district social service offices provide free intervention services to people who are economically disadvantaged and because these services are located within poor communities, these general social services may help bridge the affordability and accessibility barriers that hamper substance abuse treatment entry for people from disadvantaged communities in the Western Cape [3]*.* This may be especially true for people from the more rural parts of the province where the availability of specialist substance abuse treatment services is limited [6]. This explanation is supported, in part, by our finding that close to two-thirds of the substance-using clients attending social welfare services resided in rural districts.

Third, we found significant differences in patterns of substance use between clients attending social welfare services and clients attending specialist treatment services. Specifically, a significantly greater proportion of clients attending social welfare services compared to clients at specialist treatment centres reported alcohol as their primary substance of abuse. The prevalence of alcohol-related problems among clients attending social welfare services is not altogether surprising given the consistently high levels of alcohol-related problems reported in the Western Cape [1,2]. This is especially true for rural communities [19] which were well-represented in the SASS system. In contrast, alcohol is probably not well-represented within the specialist substance abuse treatment sector due to the primacy of methamphetamine-related problems in this sector. Methamphetamine-related problems are often associated with more acute mental health and health consequences than alcohol and as such people with methamphetamine problems enter substance abuse treatment more quickly than those with alcohol-related problems [14]. Some support for this explanation is provided by the finding that a significantly greater proportion of clients attending specialist treatment facilities cited methamphetamine as their drug of choice relative to clients in the social welfare system. Regardless of the reason for the focus on treating methamphetamine (and other illicit drugs) within the substance abuse treatment system, our findings clearly show that alcohol use remains problematic within the Western Cape and service planners and policy makers should be aware of the need for more interventions to address alcohol use in this region.

Although a significantly smaller proportion of clients reported methamphetamine use than clients within substance abuse treatment facilities, this does not mean that methamphetamine use should be neglected within general social services. Our findings point to a considerable number of participants who reported problems related to the use of methamphetamine. This highlights the importance of screening all social welfare clients for the use of methamphetamine, particularly as people using this drug were significantly more likely to report severe health, family and financial problems compared to people who used other substances. This finding has implications for the delivery of social services, because if left untreated, it is quite likely that clients using these substances will place a considerable burden on an already taxed social welfare system in the province. Social workers in district social service offices therefore need to be trained to respond and intervene effectively with people who use methamphetamine.

While our findings provide insight into how social welfare services are a point of entry into treatment and a point of intervention for people with substance use disorders, findings should be interpreted in the light of some limitations. First, the study did not utilise standard clinical screening tools such as the Alcohol Use Disorders Identification Test (AUDIT) [20] or the The Alcohol, Smoking and Substance Involvement Screening Test (ASSIST) [21] that provide cut-off scores for hazardous or harmful substance use or need for treatment. While we used frequency of substance use as a proxy measure for severity, this is a crude indicator of substance abuse severity. As such, it is difficult to assess whether the interventions provided to people were appropriate for their level of problem severity or to assess treatment need. Future research should consider using validated screening tools that assess for degree of substance use involvement and problem severity. Secondly, this study was not able to unpack and describe the causal relationships between specific substances of abuse and associated consequences or problems. Future research should include longitudinal prospective studies that track participants over time. These studies will allow researchers to determine the direction of this relationship.

## Conclusions

Despite some limitations, this study provides good evidence that social service offices are a point of entry into the substance abuse treatment sector and a point of intervention for people with substance use problems from poor communities in the Western Cape province of South Africa. In addition, findings suggest that these social service offices, conveniently located in each major district of the province, help people from poor communities overcome many of the documented structural barriers to accessing care for substance use disorders. Should policy makers and service planners continue to invest in the provision of low threshold interventions, case management and referral services at district social welfare offices; this will go some way towards improving access to services for people from underserved poor and rural communities. In addition, through triangulating findings from the SASS and SACENDU databases, we were able to compare the profile of people with substance-related problems seeking treatment in the general social service and the specialist substance abuse treatment sectors, respectively. While similarities were noted, we found that social welfare services tended to serve a more vulnerable and disadvantaged population than specialist drug treatment services. This is probably due to the fact that the location and low cost of these services overcomes many of the barriers to drug treatment entry experienced by disadvantaged communities. We also noted that more people with alcohol-related problems presented for assistance in the general social service sector than in the specialist drug treatment sector, which is probably due to the limited number of treatment slots and the pressure to treat people with methamphetamine-related problems in the latter. One recommendation is that methamphetamine use continues to be carefully monitored in the social service sector due to the severe health and social problems associated with the use of this drug. This coupled with the finding that a large proportion of people who use general social services in the province report substance use points to the urgent need to train generalist social workers in effective ways of intervening with people who use alcohol and other drugs.

## Competing interests

The authors declare that they have no competing interests.

## Authors’ contributions

NHB managed this surveillance system, analysed the data and contributed to writing the manuscript; SD contributed to data collection, analysis and the writing of the manuscript; BM conceived the study, participated in its design and coordination, and contributed to the writing of the manuscript. All authors read and approved the final manuscript.
